# Route simulations, compass mechanisms and long-distance migration flights in birds

**DOI:** 10.1007/s00359-017-1171-y

**Published:** 2017-05-12

**Authors:** Susanne Åkesson, Giuseppe Bianco

**Affiliations:** Centre for Animal Movement Research, Department of Biology, Lund University, Ecology Building, 223 62 Lund, Sweden

**Keywords:** Route simulations, Magnetoclinic route, Sun compass route, Magnetc loxodrome, Geographic loxodrome

## Abstract

Bird migration has fascinated humans for centuries and routes crossing the globe are now starting to be revealed by advanced tracking technology. A central question is what compass mechanism, celestial or geomagnetic, is activated during these long flights. Different approaches based on the geometry of flight routes across the globe and route simulations based on predictions from compass mechanisms with or without including the effect of winds have been used to try to answer this question with varying results. A major focus has been use of orthodromic (great circle) and loxodromic (rhumbline) routes using celestial information, while geomagnetic information has been proposed for both a magnetic loxodromic route and a magnetoclinic route. Here, we review previous results and evaluate if one or several alternative compass mechanisms can explain migration routes in birds. We found that most cases could be explained by magnetoclinic routes (up to 73% of the cases), while the sun compas s could explain only 50%. Both magnetic and geographic loxodromes could explain <25% of the routes. The magnetoclinic route functioned across latitudes (1°S–74°N), while the sun compass only worked in the high Arctic (61–69°N). We discuss the results with respect to orientation challenges and availability of orientation cues.

**Electronic supplementary material:**

The online version of this article (doi:10.1007/s00359-017-1171-y) contains supplementary material, which is available to authorized users.

## Introduction

Migratory birds regularly perform migrations across the globe, covering substantial distances during long continuous flights (e.g., Gill et al. 2009; Klaassen et al. 2011; Åkesson et al. 2012, 2016). Bird migration routes may evolve in response to environmental factors such as topography, availability of stop-over sites, favourable wind patterns and orientation cues (Alerstam et al. 2003), and the migration performance itself involve adaptations to flight, orientation, timing, and fuelling in individual birds (Åkesson and Hedenström 2007). The geometry of global routes followed by migratory birds has been evaluated with respect to distances and courses, for which two major types of routes have been highlighted, i.e., orthodromes and loxodromes (Imboden and Imboden 1972; Gudmundsson and Alerstam 1998). The orthodrome route corresponds to the great circle route, i.e., the shortest-distance route between two points on the globe, which followed needs continuous changing of directions along the path. The loxodrome (rhumbline) route on the other hand is slightly longer and is generated, while a constant geographic course is kept throughout the route, assuming a more simplified orientation mechanism. Both routes have been considered in studies evaluating alternative compass routes in migratory birds (e.g., Alerstam et al. 2001; Muheim et al. 2003; Grönroos et al. 2010; Åkesson and Bianco 2016).

Based on cage experiments, several compasses have been described in birds which rely on information from the sun and the related pattern of skylight polarization, stars, and the geomagnetic field (Able 1980; Emlen 1975; Wiltschko and Wiltschko 1995; Åkesson et al. 2014). The sun compass including a time-compensation mechanism, by which the birds are able to gradually correct for the apparent movement of the sun across the sky throughout the day corresponding roughly to a shift of 15° per hour of the sun azimuth relative to the horizon at high latitudes (Kramer 1950, 1952; Schmidt-Koenig 1958, 1990). The star compass, on the other hand, provides a direction relative to the rotation centre of the night sky, i.e., geographic north, without a time-compensation mechanism (Emlen 1967, 1970). The geomagnetic field provides a globally available source of information, which may be explored by migratory birds for compass orientation and navigation (Wiltschko and Wiltschko 1995, 2009). The magnetic compass of birds is dependent on detection of the angle of inclination, i.e., the angle by which the geomagnetic field lines cross the surface of the earth, and not the polarity of the geomagnetic field (Wiltschko and Wiltschko 1972), providing a tool to differentiate directions along a north–south axis leading towards the poles or the equator. Young birds have an inherited capacity to explore the alternative compasses, but they need to experience a combination of natural compass information during ontogeny to use the information for compass orientation (Able and Able 1996; Emlen 1975), and establish a population-specific orientation (Weindler et al. 1996). Compasses may further be recalibrated during migration (Able and Able 1995; Cochran et al. 2004; Muheim et al. 2006; cf. Åkesson et al. 2015). Despite substantial accumulated knowledge of compass mechanisms and calibration processes, we still do not know exactly what and how compass/-es are used during active migration flights. However, the question has been approached by predicting flight routes based on alternative compass mechanisms (Kiepenheuer 1984; Alerstam and Pettersson 1991).

Recent development of tracking technology provides new possibilities to investigate avian compass orientation and navigation based on field tracking data (Guilford et al. 2011). Here, we evaluate on the basis of published route evaluations using simulations and data collected from different field studies, what support is available for alternative compass mechanisms activated during long migration flights. To make the set of alternative compass routes complete, we have generated by simulations magnetoclinic routes (Kiepenheuer 1984) for cases which did not include such routes in the original evaluations (Alerstam et al. 2001; Muheim et al. 2003; Grönroos et al. 2010). We based our simulations on the same parameters given in the previous work making the routes strictly comparable with the previously published data (Supplementary material, Fig. S1–S5). Based on a complete set of routes, we thereafter explore if there were any differences in what compass may be used depending on geographical area, and discuss outcomes relative to available cues. We further discuss what challenges birds face in using the alternative compasses depending on where on the globe they are, and what environmental factors may change their ability to keep selected courses. Our ultimate goal is to investigate if, based on present route simulations, we can identify a functional compass mechanism that may be used by birds during long-distance migration flights in different parts of the globe, or if we find support for alternative compasses depending on geographic region. We hypothesize that all species of birds can use a time-compensated sun compass (Kramer 1950, 1952; Schmidt-Koenig 1958, 1990), stars (Emlen 1975), and the geomagnetic field (Wiltschko and Wiltschko 1972) for compass orientation. Here, we do not consider the ability to navigate using a map (Kramer 1957), since we would like to keep the different studies strictly comparable, as data on routes and directions recorded are based on observations from both juvenile and adult birds, or a combination of both, for which the ability to navigate may be questioned in juvenile birds (e.g., Perdeck 1958).

### Compass mechanisms and migration routes

**Fig. 1:**
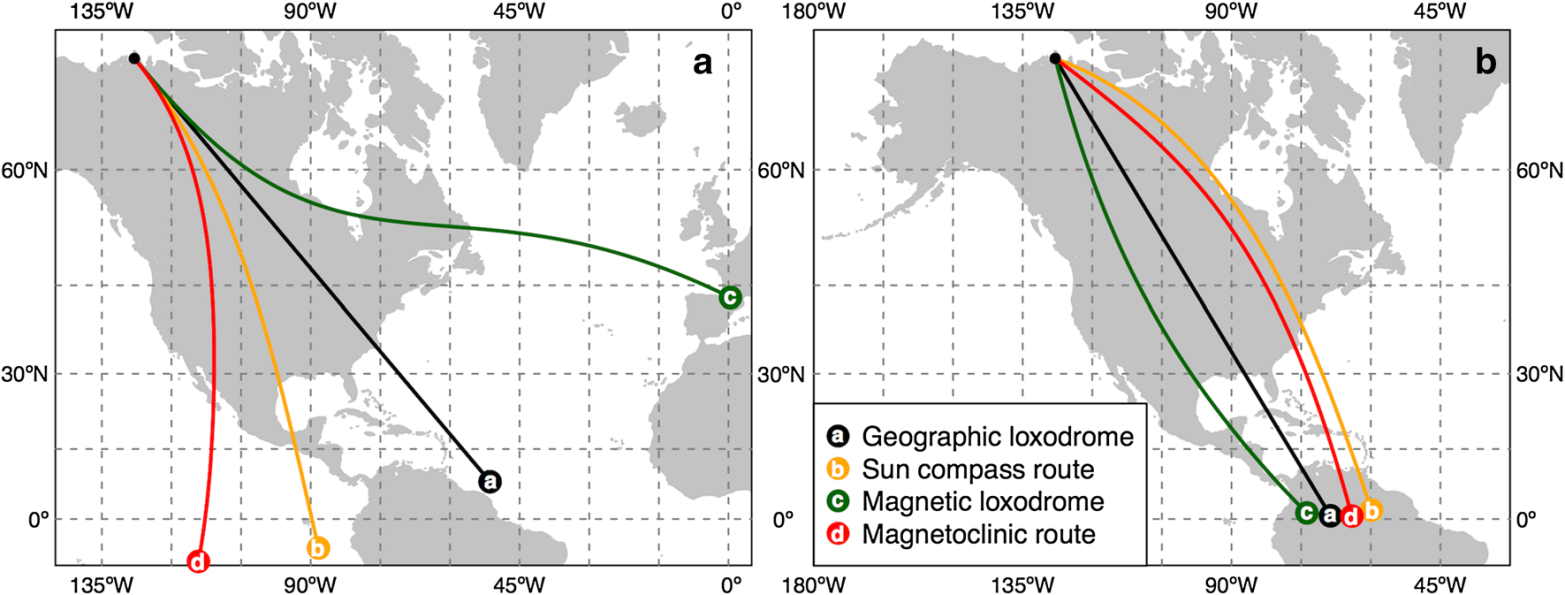
Alternative compass routes based on compass information from the stars, the sun, and the geomagnetic field. Routes are for the autumn migration from north-west Canada (*solid black dot*; 70°N, 128°W) to equatorial South America. All routes are 9000 km long with **a** same initial direction (140°) and **b** specific direction for each compass mechanism that resulted in a successful route (geographic loxodrome = 149°; sun compass = 110°; magnetic loxodrome = 165°; magnetoclinic route = 126°). If a compass mechanism is used for the entire route, small differences in the initial direction may result in dramatic differences in the destination location. Maps are in Mercator projection (with 15° grid), so that the geographic loxodrome route (with constant geographic direction) is represented as *strait line*

All three biological compasses based on the sun, the stars, and the geomagnetic field (Able 1980; Emlen 1975; Wiltschko and Wiltschko 1995; Åkesson et al. 2014) may generate predictable routes on the basis of the mechanisms described (Fig. 1). Loxodromes following constant geographic courses may be followed based on knowledge of geographic north by rotating stars at night (Emlen 1967, 1970), or the rotation centre of the daytime sky using information from the sun and the skylight polarization pattern (Fig. 1). The orthodromic route, as mentioned above, needs continuous course changes, and may theoretically be approximated by shorter loxodromes, i.e., fixed course flights (as described above), but each flight with a new direction. Alternatively, an orthodromic route may be reached by use of a time-compensated sun compass without changing the internal physiological time to the local time during flights as proposed by Alerstam and Pettersson (1991; Fig. 1). Alerstam et al. (2001), based on tracking radar data, found support for sun compass routes corresponding approximately to orthodromes followed by migrating waders in the high arctic.

Magnetic loxodromes, i.e., fixed courses relative to geomagnetic north, may be followed based on detection of the alignment of the geomagnetic field (Wiltschko and Wiltschko 1972; Fig. 1). As an alternative, Kiepenheuer (1984) proposed that birds may follow a magnetoclinic route using the angle of inclination of the geomagnetic field (Wiltschko and Wiltschko 1972; Fig. 1). According to this principle, the birds will follow the apparent angle of inclination, and keep a fixed heading relative to the angle of inclination along the route. Kiepenheuer (1984) pointed out that birds orienting by this principle will fly along curved routes, largely corresponding to natural migration routes found in the northern Hemisphere, since curved routes will be formed when they move south (Fig. 1; cf. also Supplementary material).

### When and where do we find challenges using celestial and geomagnetic compasses?

Birds have been able to disperse and explore most parts of the globe during breeding, migration, and wintering. Sometimes, birds perform long continuous flights lasting for several days to reach distant areas (e.g., Gill et al. 2009; Klaassen et al. 2011; Åkesson et al. 2016). However, if we consider how migratory birds may be able to use compass information from the sun and the skylight polarization pattern, stars, and the geomagnetic field, we find challenges associated with geographical regions, but also related to environmental conditions such as geomagnetic storms, forest fire smoke, cloud cover, and fog. Below, we discuss the types of problems that birds face on natural migrations in more detail, and what effect they may have on the use of different compasses.

### Polar regions

The most challenging areas for compass orientation and navigation are perhaps the polar regions, since here, we find either continuous daylight in the polar summer or continuous darkness in the polar winter with limited variations in light level throughout the day and steep geomagnetic field lines. Polar regions are, furthermore, often covered by fog and clouds due to frequent passages of low-pressure systems, and variations in sea surface temperatures that may lead to limitations to detect celestial information as well as the occurrence of specific optical phenomena that may be used for navigation at a distance (Hegedüs et al. 2007a, b, c, d). The availability of celestial information for orientation, thus, varies largely with season and weather. In summer, the high arctic tundra is inhabited by long-distance migrating waders, geese, and passerines exploring the rich areas for breeding. At this time of year, the continuous daylight results in limited possibility to see and learn to use a rotating star pattern by young birds born in this area, which is crucial for orientation in young birds (Emlen 1975; Weindler et al. 1996). Stars are visible in a night sky only when the sun has reached −6° below the horizon (Åkesson et al. 1996). Continuous daylight may further cause the internal time sense to drift across time (e.g., Gwinner 1977, 1990; Gwinner and Benzinger 1978; Hau and Gwinner 1994; cf. Krüll 1976), which in turn may result in problems using the time-compensated sun compass (Kramer 1950, 1952; Schmidt-Koenig 1958, 1990).

**Fig. 2:**
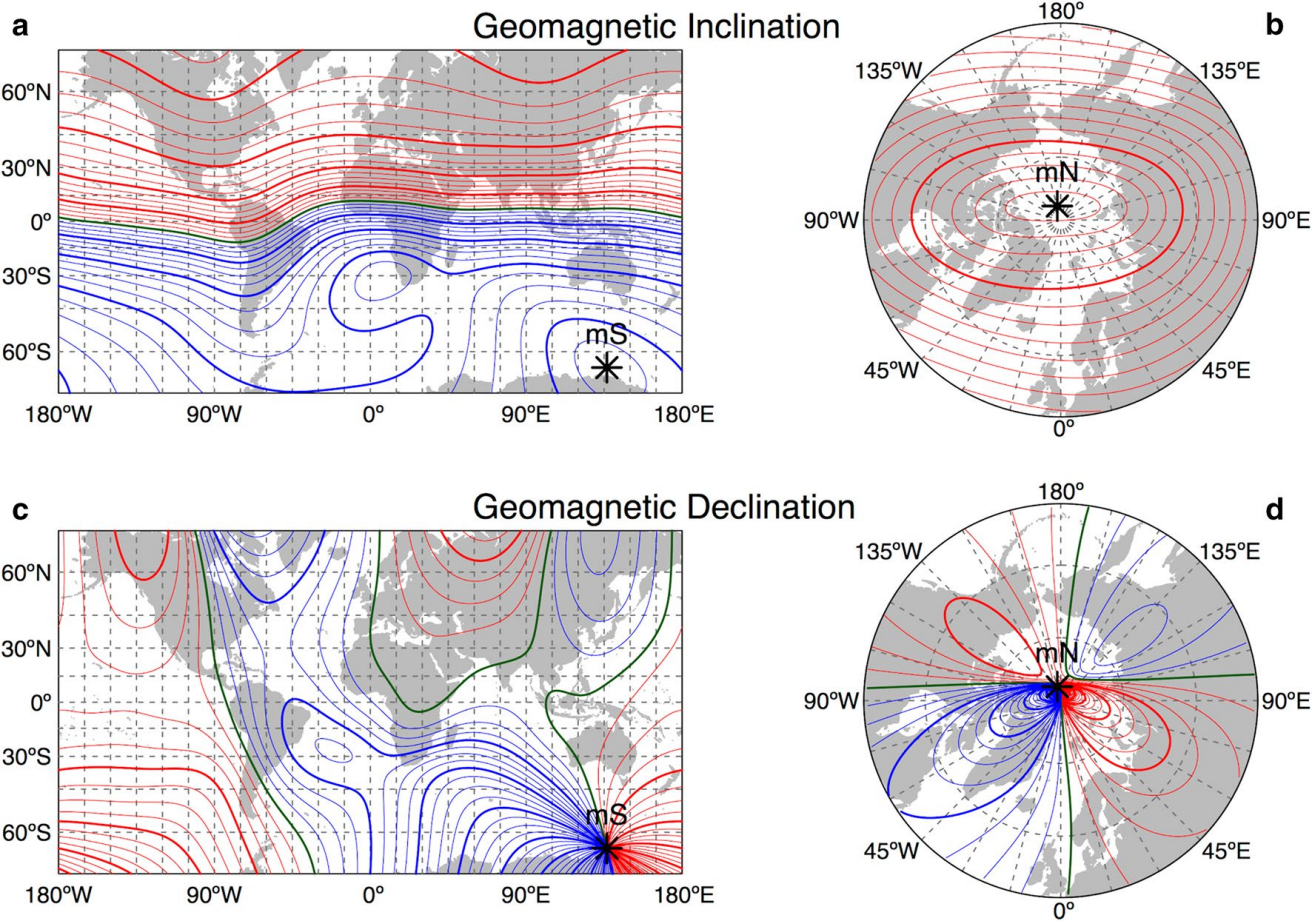
Map of the geomagnetic gradients of the angle of inclination (**a, b**) and declination (**c**, **d**). Isolines are reported in *red* for positive values, in *blue* for negative values, and in *green* for the 0°. *Thicker lines* are separated by 20° and *thinner lines* by 5° (**a**, **c**, **d**) or 2° (**b**). Maps are reported in Mercator projection (**a** and **c** for 70°S—70°N) and Gnomonic projection (**b** and **d** for 50°N—90°N) with 15° grid. The position of the magnetic south (mS) and magnetic north (mN) is also reported. The geomagnetic field was obtained for the year 2015 using the 12th generation of the International Geomagnetic Reference Field model (IGRF-12; see also Supplementary material)

Birds inhabiting the high arctic experience naturally very steep geomagnetic field lines which may make the geomagnetic field challenging to use because of problems detecting the inclination (Fig. 2; Wiltschko and Wiltschko 1972; cf. Sandberg et al. 1998; Åkesson et al. 1995, 2001a). In the laboratory, songbirds have been shown to detect magnetic fields as steep as 85° inclination angle (Lefeldt et al. 2015), while under natural conditions, the behavioural responses suggest an ability to detect even steeper magnetic fields (Sandberg et al. 1998; Åkesson et al. 2001a, b, 2005). Polar regions are further exposed to particularly large temporal variations of geomagnetic field parameters caused by geomagnetic storms (Skiles 1985), which may be challenging for compass orientation and navigation. At high latitudes, there are large differences in declination between nearby sites when travelling across longitudes and substantial drift of geomagnetic parameters as a result of secular variation (Skiles 1985). Longitude detection is particularly challenging for navigating animals moving across the globe (Gould 2005). However, the angle of declination, i.e., angular difference between geomagnetic and geographic north, may be used at high latitudes to define longitude as here, the declination largely varies with longitudes (Fig. 2; Åkesson et al. 2005). The temporal variations of the geomagnetic field during magnetic storms, being particularly large at high latitudes (Skiles 1985), will likely make magnetic compass orientation difficult during these situations (Moore 1977).

### Equator region

Near the geographic and geomagnetic equators, the daylength varies less across the year (near to 12:12 h cycle) providing reliable and predictable circadian time-keeping (e.g., Gwinner 1986, 1990, 1996; Helm et al. 2009). However, an unobstructed view of the center of the rotating night sky including learned star patterns nearby (Emlen 1975) is not any longer available, as the altitude of the center of rotation now appears to be closer to or below the horizon for an observer located at the equator. Instead, new star patterns become available in this region, follow a simpler path across the sky rising in the east, cross near to the zenith point, and then again disappear below the horizon in due west. Stellar orientation based on experiences from high latitude breeding areas can, thus, be expected to be challenging in equator regions, and needs modifications as new stars become available and the perceived path of stars across the sky changes compared to regions further away from the equator (Emlen 1975). We may, however, assume that birds have the capacity to learn new star patterns as they become visible near the equator (Emlen 1967, 1970).

The sun’s apparent movement across the sky is further largely changed as compared to more northern latitudes, because the sun, just like stars, follows a path, where it appears above the horizon in the east at sunrise, follows a steep trajectory to the zenith point of the sky, and thereafter lowers itself to the west, where it disappears again below the horizon as the sun sets. The change of the sun’s azimuth in relation to the horizon near the equator largely differs from the approximate 15° per hour movement at high latitudes, where also the sun elevation shows much less variation across the day (Wehner 1998). An additional problem using the sun near the equator is associated with the change of the path from south of the zenith point at midday to north when the bird has moved to a location south of the equator. How migratory birds cope with the change of the movement path of the sun relative to the horizon from south to north of the zenith point as the equator is crossed, also remains to be explained. Release experiments with homing pigeons at the geomagnetic equator suggest the use of sun-related information to home from 300 km distances when released in morning and afternoon, but difficulties at midday when the sun has reached the zenith position (Ranvaud et al. 1983). Homing pigeon, though, are resident and fly across a limited area and migratory birds may, therefore, handle the sun compass in a different way as they cross latitudes on migration.

Near the geomagnetic equator, the field strength of the earth’s magnetic field is reduced to the half (<25,000 nT) as compared to polar regions (>68,000 nT), and here, the field lines align with the horizontal plane (Fig. 2; Skiles 1985). Migratory birds have been shown experimentally to adapt to reduced field strengths, suggesting that their magnetic compass is adapted to meet these challenges (Wiltschko 1978; Wiltschko and Wiltschko 1995). The avian inclination compass is impossible to use in a horizontal geomagnetic field, as birds cannot based on the magnetic field discriminate between north and south (Wiltschko and Wiltschko 1972), leading to expected problems to orient at the geomagnetic equator. Compass orientation in a naturally near horizontal geomagnetic field has been investigated in field experiments in East Africa with migratory Marsh Warblers *Acrocephalus palustris* during autumn migration, where the warblers seem to be highly dependent on celestial information for orientation at sunset after crossing the geomagnetic equator (Åkesson 1993a). However, it remains to be investigated how near the geomagnetic equator migratory birds may be able to use their inclination compass (cf. Ranvaud et al. 1983; Sandberg et al. 1998; Åkesson et al. 2001a). Experiments with birds in the laboratory suggest that they may detect angles of inclination of 5° (Schwarze et al. 2016), but the question is if they can detect even smaller angles of inclination under natural conditions as experiments in the high Arctic suggest (Sandberg et al. 1998; Åkesson et al. 2001a, 2005).

### Geomagnetic anomalies and temporal variations of the geomagnetic field

Geomagnetic anomalies are the result of magnetic rocks associated with iron deposits in the ground, which may cause the resulting local geomagnetic field to deviate largely from the global geomagnetic field generated in the center of the earth (Skiles 1985). The local geomagnetic field near to strong magnetic anomalies may result in substantial deviations of both total intensity and declination, as is the case for an anomaly located south of Moscow near Kursk (local intensity >190,000 nT; declination 60°E–110°W within <1 km; Wiltschko and Wiltschko 1995). Birds migrating across a geomagnetic anomaly in central Sweden tracked by radar have been shown to react by decreasing flight altitude as they approach the edge of the anomaly (Alerstam 1987), suggesting that they were using magnetic information for orientation during active migration flights. The strongest influence of magnetic anomalies is expected at low flight altitudes near the anomaly, and birds migrating at one-to-several thousand meters altitudes are, therefore, presumably less affected.

The strongest influence of temporal variations of the magnetic field detected on the earth caused by solar flares and sunspot activities is experienced in polar regions (Skiles 1985), as mentioned above. Magnetic daily variations, however, are also strongest may be on this side of the globe, where the sun’s rays are reaching the ground, at daytime (Skiles 1985). Migration at night will, thus, reduce the influence of temporal variations caused by the sun’s influence on the earth magnetosphere but may, as have been pointed out, also be related to explorations of reduced turbulence in the air masses (Kerlinger and Moore 1989).

### Orientation under overcast skies, forest fire smoke, and fog

It has long been assumed that celestial information is not available for orientation during overcast and foggy conditions, since birds cannot get access to unobstructed view of stars and the sun. However, in recent years, measurements of the skylight polarization pattern under total overcast and in foggy conditions have revealed that between 5 and 10% of the incoming light is polarized under these conditions with the strongest polarization visible near the horizon, and with the symmetry plane of the skylight polarization pattern remaining largely the same as under clear sky conditions (Hegedüs et al. 2007a, b). Whether birds are able to detect such low levels of polarization remains, however, to be investigated (Åkesson 2014; Åkesson et al. 2014). Forest fire smoke, potentially covering large geographical areas, will cause large deviations in the natural skylight polarization pattern leading to expected problems to use this information for compass orientation (Hegedüs et al. 2007c). Insects, largely dependent on the skylight polarization pattern for orientation (Brines and Gould 1982; Wehner 2001; Horváth and Varjú 2003), have been shown to become disoriented under forest fire smoke, presumably as a consequence of the distorted skylight polarization pattern (Hegedüs et al. 2007c). Whether birds would be affected in a similar way by forest fire smoke needs still to be investigated (cf. Alarcón et al. 2016). Nocturnal bird migrants tend to initiate migration under clear or partly overcast conditions when celestial information from stars, the sun, and the skylight polarization pattern is more readily available (Åkesson et al. 1996, 2001b; Bolshakov et al. 2007; Sjöberg et al. 2015). A clear sky, however, is often associated with favourable wind conditions for migration in Western Europe, and thus, it is hard to discriminate which of several factors are the most important for birds deciding to take-off on migration flights (Åkesson et al. 2001b; Sjöberg et al. 2015).

## Route simulations

**Fig. 3:**
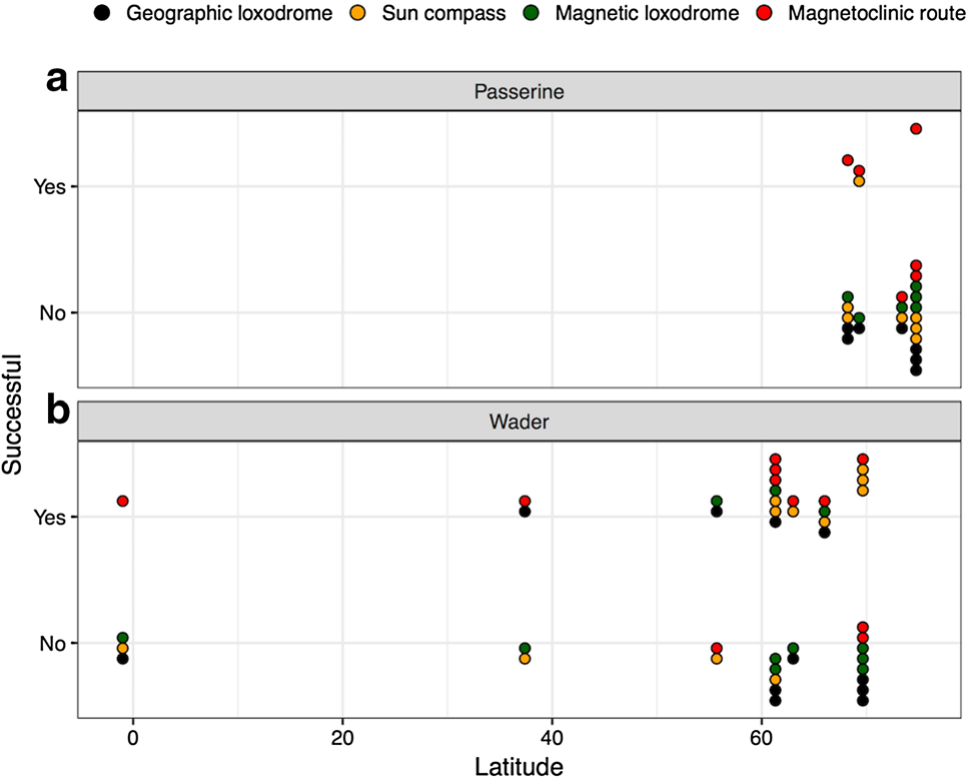
Overview of results from route simulations based on the different compass mechanisms: geographic loxodrome, sun compass route, magnetic loxodrome, and magnetoclinic route. The outcome (yes, no) is plotted relative to the starting latitude of the simulations for **a** Passerines and **b** waders, respectively

Here, we were able to generate missing magnetoclinic routes in previous studies (Alerstam et al. 2001; Muheim et al. 2003; Grönroos et al. 2010), so that we could evaluate the four alternative routes based on magnetic and celestial compass mechanisms for all studies (Table 1). A successful route means that it leads the bird near to the expected destination area. For all routes (i.e., magnetoclinic, sun compass, and magnetic and geographic loxodromes) simulated by previous studies (Alerstam et al. 2001; Muheim et al. 2003; Grönroos et al. 2010; Åkesson and Bianco 2016) and complemented by us in this study (*n* = 18; Table 1), we found that the magnetoclinic route was successful in more than half of the considered cases (65%), while the second best the sun compass route was successful in less than half the cases (44%; Fig. 3). The geographic and magnetic loxodromes were valid only in 1/5 of all simulated scenarios (22 and 18%, respectively). However, for these evaluations, both displaced birds and birds performing natural migrations were combined, as well as orientation recorded by different methods.

**Table 1:** Evaluation of migration routes based on different compass mechanisms available in literature

Taxon	Species	Latin name	Season	Region	Start location		Route success				References	Orientation data			
					Geographic	Lat	Geographic loxodrome	Sun compass	Magnetic loxodrome	Magnetoclinic route		Type	References	*N*	Age class
Wader	Bar-tailed Godwit	*Limosa lapponica*	Autumn	Pacific (Alaska-Oceania)	Alaska	61.4	Yes	Yes	Yes	Yes	Åkesson and Bianco (2016)	Satellite telemetry	Gill et al. (2009)	9	–
Wader	Great snipe	*Gallinago media*	Autumn	Europe-Africa	Jämtland (Sweden)	63	No	Yes	No	Yes	Åkesson and Bianco (2016)	Geolocator	Klaassen et al. (2011)	3	Adult
Wader	Great snipe	*Gallinago media*	Spring	Europe-Africa	Central Africa	−1	No	No	No	Yes	Åkesson and Bianco (2016)	Geolocator	Klaassen et al. (2011)	3	Adult
Wader	Bar-tailed Godwit	*Limosa lapponica*	Spring	Europe	Lund (Sweden)	55.7	Yes	No	Yes	No	Åkesson and Bianco (2016)	Tracking radar	Green (2004)	14	Adult
Passerine	Northern wheatear	*Oenanthe oenanthe*	Autumn	North Atlantic (Greenland-Africa)	Disko Island (Greenland)	69.3	No	Yes	No	Yes	Åkesson and Bianco (2016)	Geolocator	Ottosson et al. (1990)	11	Juvenile
Wader	Whimbrel	*Numenius phaeopus*	Spring	North America	Delmarva Peninsula	37.39	Yes	No	No	Yes	Åkesson and Bianco (2016)	Satellite telemetry	Watts et al. (2008)	1	Adult
Wader	Sharp-tailed sandpiper	*Calidris acuminata*	Autumn	Pacific (Alaska-Oceania)	Kanaryarmiut Field Station	61.21	No	Yes	No	Yes^a^	Grönroos et al. (2010)	Orientation cage (overcast)	Grönroos et al. (2010)	23	Juvenile
Wader	Sharp-tailed sandpiper	*Calidris acuminata*	Autumn	Pacific (Alaska-Oceania)	Kanaryarmiut Field Station	61.21	No	No	No	Yes^a^	Grönroos et al. (2010)	Orientation cage (clear skies)	Grönroos et al. (2010)	27	Juvenile
Passerine	Savannah sparrow	*Passerculus sandwichensis*	Autumn	North America	Inuvik	68.21	No	No	No	Yes^a^	Muheim et al. (2003)	Orientation cage	Muheim and Åkesson (2002)	17	Juvenile
Passerine	White-crowned sparrow	*Zonotrichia leucophrys*	Autumn	North America	Inuvik	68.21	No	No	n.a.	n.a.	Muheim et al. (2003)	Orientation cage	Åkesson et al. (2001a), b)	77	Adult, juvenile
Passerine	White-crowned sparrow	*Zonotrichia leucophrys*	Autumn	North America	Banks Island	73.39	No	No	No	No^a^	Muheim et al. (2003)	Orientation cage (displaced)	Åkesson et al. (2001a), b)	26	Adult, juvenile
Passerine	White-crowned sparrow	*Zonotrichia leucophrys*	Autumn	North America	Melville Island	75.07	No	No	No	No^a^	Muheim et al. (2003)	Orientation cage (displaced)	Åkesson et al. (2001a), b)	26	Adult, juvenile
Passerine	Snow bunting	*Plectrophenax nivalis*	Autumn	North America	Resolute (Cornwallis Island)	74.41	No	No	No	No^a^	Muheim et al. (2003)	Orientation cage	Sandberg et al. (1998)	9	Juvenile
Passerine	Snow bunting	*Plectrophenax nivalis*	Autumn	North America	Resolute (Cornwallis Island)	74.41	No	No	No	Yes^a^	Muheim et al. (2003)	Release experiment	Sandberg et al. (1998)	24	Juvenile
Wader	(Multiple species)	–	Autumn	North-Central America	Beufort Sea	70	No	Yes	No	No^a^	Alerstam et al. (2001)	Tracking radar	Alerstam et al. (2001)	–	–
Wader	(Multiple species)	–	Autumn	North-Central America	Wollaston Peninsula	69.3	No	Yes	No	No^a^	Alerstam et al. (2001)	Tracking radar	Alerstam et al. (2001)	–	–
Wader	(Multiple species)	–	Autumn	North-Central America	King William Island	69.4	No	Yes	No	Yes^a^	Alerstam et al. (2001)	Tracking radar	Alerstam et al. (2001)	–	–
Wader	(Multiple species)	–	Autumn	North-Central America	Baffin Island	66	Yes	Yes	Yes	Yes^a^	Alerstam et al. (2001)	Tracking radar	Alerstam et al. (2001)	–	–

A successful route is the one that leads the bird near to the expected destination area. Two routes are not available (n.a.), because orientation data were not provided. Registration method, number of birds, and age class are given for the orientation data used for route simulations

^a^Route simulation performed in this study and available as Supplementary material

We noted that for only 3 out of 18 cases, the routes have been simulated for spring migration starting at lower latitudes (1°S–55.7°N; Table 1), and for those cases, the geographic loxodrome and the magnetoclinic route were successful twice, while the sun compass was never successful (Fig. 3). The magnetic loxodrome was successful only once in spring out of the three simulated cases (Table 1; Fig. 3).

Routes were simulated for both passerines (*n* = 7 for geographic loxodrome and sun compass; *n* = 6 for magnetic loxodrome and magnetoclinic routes) and waders (*n* = 11). For passerines, the magnetoclinic route was valid in 50% of the cases, but only in one case (14%), the sun compass route was successful. We found that a geographic or magnetic loxodrome was never able to explain the orientation for passerine migrants (Table 1; Fig. 3). For waders, the most successful routes were the magnetoclinic (73%) and thereafter the sun compass (64%), with only a few successful cases for the geographic (36%) and magnetic (27%) loxodrome routes (Table 1; Fig. 3).

We found that in only two cases, all considered compass mechanisms were successful in explaining the routes followed, for the case of the Bar-tailed Godwit crossing the Pacific Ocean (Gill et al. 2009; routes evaluated in Åkesson and Bianco 2016) and the waders heading to South America from Baffin Island in northern Canada (Alerstam et al. 2001) (Supplementary material, Fig. S5).

There were only few cases for passerines (White-crowned Sparrow, Åkesson et al. 2001a, b; Snow Bunting, Sandberg et al. 1998) for which none of the compass mechanism resulted in a successful route (Muheim et al. 2003 and Supplementary material, Figs. S3 and S4). However, from two of the sites with route simulations, the birds have been displaced north of the breeding range, and therefore, the resulting routes may be treated with caution. If we exclude that the route simulations generated based on orientation cage experiments with displaced birds, the support for the use of a magnetoclinic route become even stronger (73% out of 15 simulations). The same is true for a sun compass route receiving stronger support excluding the displaced birds (50% out of 16 simulations).

In 10 out of 18 cases (56%; including the displaced White-crowned Sparrows; Åkesson et al. 2001a, b), the route simulations were considered from the autumn migration from North America (either Alaska or arctic Canada) for both passerines and wader species (Table 1). For this system, sun compass and magnetoclinic routes were successful in four cases each (40%), and geographic and magnetic loxodrome only in one case (with start location from Baffin Island; Alerstam et al. 2001). For the spring migration in North America, only one case was considered (Whimbrel, *Numenius phaeopus*) and only the two alternative geomagnetic routes (magnetic loxodrome and magnetoclinic route) were resulting in successful routes (Table 1; Åkesson and Bianco 2016).

The extreme endurance wader migration across the Pacific from Alaska to New Zealand and Australia forms a challenge to birds which cannot land, but are forced to fly continuously for more than 1 week. These flights are ideal for compass route simulations as effects of landmarks on orientation can be excluded for major parts of the route. For the autumn migration across the Pacific Ocean by the two wader species (Bar-tailed Godwit, Gill et al. 2009, and Sharp-tailed Sandpiper, Grönroos et al. 2010), the magnetoclinic route was successful on all three cases (100%), the sun compass in two (66%), and the geographic and magnetic loxodrome in one each (33%).

In the examples for which routes have been simulated based on tracking data for the Palaearctic-African migration system, the outcome shows that the magnetoclinic route was successful in two out of three cases (66%) and the other three compass mechanisms in only one case each (33%). In the unique case of the Northern Wheatear *Oenanthe oenanthe* migrating from Greenland to western Africa across the ocean, sun compass and magnetoclinic routes were the only two possible options that would generate realistic routes across the North Atlantic leading to predicted destinations in southwestern Europe and Africa (Table 1; Åkesson and Bianco 2016). We find this species of particular interest and will discuss the Northern Wheatear migration from the most eastern part of its breeding range in more detail below.

### The special case of the Northern Wheatear

**Fig. 4:**
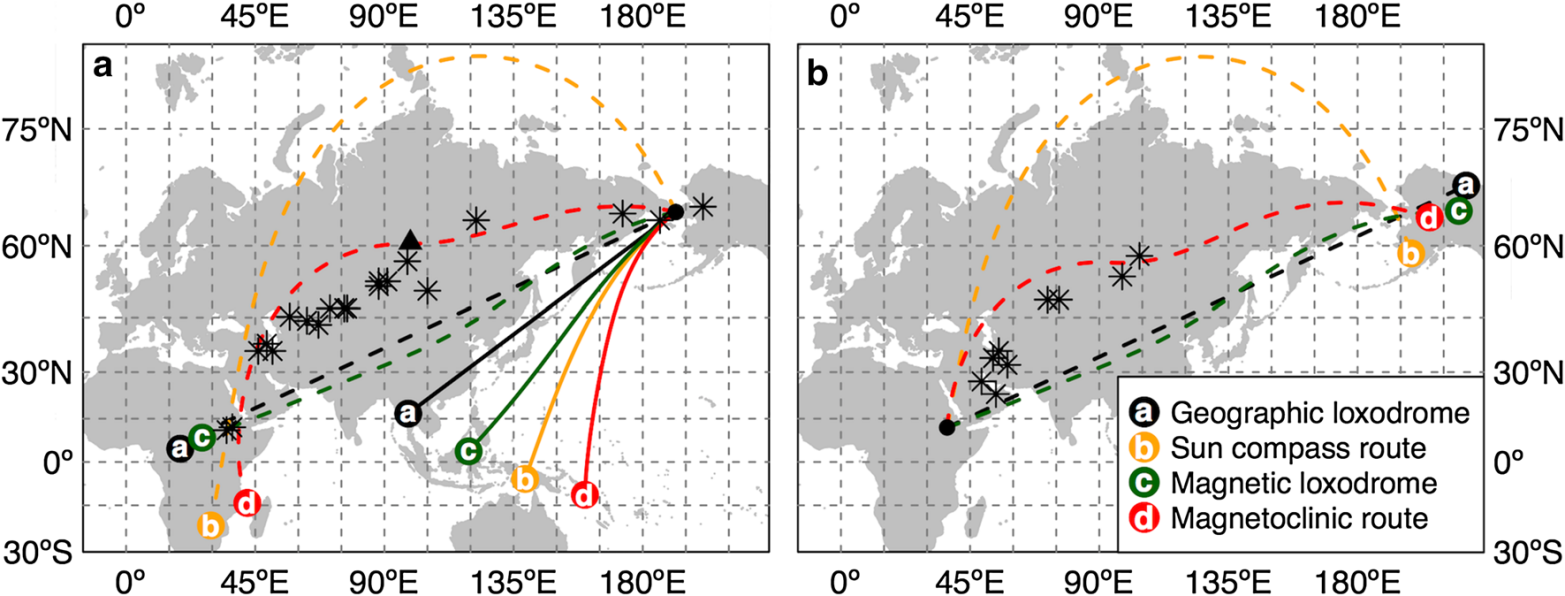
Northern Wheatear simulated routes using alternative compass mechanisms for **a** autumn migration and **b** spring migration. Routes reported as solid lines (**a** 9000 km) refer to departure directions from radio telemetry tracking (Schmaljohann et al. 2013). Routes reported as *dashed lines* (**a, b**) are examples or successful migratory route for each compass mechanism. *Dashed lines* are 15,000 km long except for **b**, magnetoclinic route (13,000 km) and sun compass route (12,000 km). *Black stars* represent stop-over locations, for both autumn and spring, determined with light-based geolocators tags (Schmaljohann et al. 2012). According to Schmaljohann et al. (2012), the magnetoclinic route is the only compatible with stop-over locations (it is closer to stop-over locations in both seasons), but during autumn, migration requires a change in direction along the migration (reported in **a** as *solid black triangle*). Routes in autumn and spring have slightly different shapes, because we did not consider the same destination location for all the compasses, but rather, a wider region compatible with wintering and breeding areas for this species. Maps are in Mercator projection with 15° grid. A special case for the spring migration along the magnetoclinic route is reported in Supplementary material, Fig. S6

The Northern Wheatear has an almost completely circumpolar distribution, breeding on high arctic tundra and wintering in sub-Saharan Africa, for which some populations are known to perform the longest passerine migration on earth (Conder 1989). An extremely challenging case for bird navigation is the 30,000 km long round-trip migration of the Northern Wheatear from Alaska to eastern Africa and back (Bairlein et al. 2012; Schmaljohann et al. 2012, 2013). Such a long migration lasts up to 3 months and requires the crossing of several meridians and ecological barriers (deserts, seas, and high mountains). Based on radiotelemetry tracks of juvenile birds, Schmaljohann et al. (2013) showed that the departing direction in autumn from the westernmost location of mainland Alaska was not compatible with any compass mechanism that could lead the birds to their wintering area in eastern Africa. Indeed, such orientation would carry the birds southwest towards the Indian Ocean or Australia (Fig. 4a). On the other hand, Schmaljohann et al. (2012) tracked adult individuals, during both autumn and spring migration, using light-level loggers and compared their individual tracks with different compass courses. They concluded that the only possible route that could explain what the birds were doing during natural migrations was the magnetoclinic route (Fig. 4, modified from Schmaljohann et al. 2012).

Although the shortest route for the Northern Wheatear breeding in Alaska is represented by a sun compass route approximating an orthodrome (cf. Schmaljohann et al. 2012), such a route would lead the bird to cross the Arctic Ocean at very high latitudes without favourable locations to stop (Fig. 4). The crossing of inhospitable and challenging areas, however, would not be the only limit for a sun compass course. Indeed, such a small bird will require frequent stopovers to refuel lasting at least 5 days (Schmaljohann et al. 2012). A stop lasting three or more days would further reset the bird’s internal clock (Schmidt-Koenig 1990), such that it would not be able to strictly follow a sun compass route as outlined by Alerstam and Pettersson (1991). By enjoying repeated stopovers, the Northern Wheatear would rather be predicted to follow a loxodrome. A geographic loxodrome, and for this matter also a magnetic loxodrome, cannot be considered successful, because they are not compatible with the routes recorded by geolocators as shown by Schmaljohann et al. (2012), since they align much further south across China and India (Fig. 4a).

The only possible migration route for both the autumn and the spring for the Northern Wheatear migrating between Alaska and eastern Africa is, thus, represented by a magnetoclinic route sensu Kiepenheuer (1984). The magnetoclinic route is similar in shape and distance to real migration tracks, where stop-over sites have been located north of the Himalayas (cf. Schmaljohann et al. 2012; Fig. 4). However, the autumn migration along the magnetoclinic route requires an additional assumption that is that the bird should initially follow the isoclinic line of the departing location in Alaska in a westerly direction, and only after a considerable longitudinal displacement, the bird should change its heading towards south (Kiepenheuer 1984). In our example simulation, we assumed that the bird changed from an apparent inclination angle of 75.4° (following the departure isocline) to 76° (heading southward) after 4600 km from the departure in autumn (solid black triangle in Fig. 4a). Kiepenheuer (1984) speculated that probably, the massif of the Himalayan Mountain prevents the bird to head southward too early and predicted more northerly routes for the Alaskan wheatears, which has now been confirmed by geolocator tracking data (Bairlein et al. 2012; Schmaljohann et al. 2012).

A successful magnetoclinic route during spring migration would not require any additional course change, but would be successful just keeping an apparent inclination angle equal to the local geomagnetic inclination of the destination area in Alaska (around 76°; Fig. 4a). However, a strict magnetoclinic route will not be compatible with the known stop-over locations at the Arabic Peninsula and in the south of the Caspian Sea (Schmaljohann et al. 2012; Fig. 4a). One possibility in this case is that the wheatears depart along the magnetoclinic route only from the stop-over location at the Arabic Peninsula (Supplemental material, Fig. S6). This hypothesis would imply an intermediate goal strategy (Rabøl 1978) that requires an external cue to trigger the change in compass orientation, possibly involving a map sense (Kramer 1957). It is interesting to note that the locations where a change in compass course would be required, both in autumn (Fig. 4a) and in spring (Fig. S6), are located very close to stop-over sites explored by Northern Wheatears on migration as has been confirmed by tracking data (Bairlein et al. 2012, Schmaljohann et al. 2012).

## Discussion

### Which compass mechanism is used during migration flights?

Our aim was to identify, if possible, a compass mechanism that may be at work during active long migration flights performed by birds in different geographical regions. We must, however, conclude that we were not able to identify one single mechanism that found support by our recent and previous route simulations at all sites (Table 1, Alerstam et al. 2001; Muheim et al. 2003; Grönroos et al. 2010; Åkesson and Bianco 2016, this study), without also providing some support for other alternative mechanisms. The compass mechanism that in a majority of cases was supported by real tracking data and through the data used from migratory departure directions recorded by different methods (Table 1) was the magnetoclinic route proposed by Kiepenheuer (1984). The magnetoclinic mechanism could explain the trajectories for 65% of all routes, and even up to 73% of the cases excluding experiments with displaced birds. The sun compass route, meaning that birds in active flight would use their time-compensated sun compass, but not compensate for local time shifts along the route (Alerstam and Pettersson 1991), was the second best mechanism explaining 44% of all routes, and 50% of the routes when displaced birds were excluded.

We found, however, some problematic regions for both these compasses, for which the magnetoclinic route met the strongest challenges in the arctic at extremely high latitudes, leading in this situation to routes that spiralled north approaching the magnetic North Pole, rather than deviating gradually to the south as expected in autumn (e.g., Supplemental material Fig. S5, locations 1 and 2). Some of those simulations, however, were based on data from displaced birds and may, therefore, be treated with caution. Indeed, the apparent inclination angle is defined only for values larger than the local inclination angle and 90° (Kiepenheuer 1984). As a consequence, when the local inclination is very steep and closer to 90°, the geographic directions compatible with a valid apparent inclination angle are limited, and sometimes, the solution do not exist (Kiepenheuer 1984). A possibility in this case is that the bird follows an alternative compass or still uses the magnetic compass to head southward, and when it crosses the isocline with a lower angle of the fixed migratory apparent inclination angle, it starts to follow the magnetoclinic compass route. Furthermore, a compass based on the magnetoclinic hypothesis (Kiepenheuer 1984) would always have two solutions (towards east or west) and a secondary cue should be used to select the appropriate direction. A less obvious consequence is that two alternative migratory routes could exist, and in some cases, both could result in valid routes such as in the case shown in Fig. S1 (Supplemental material), where both south-west and south-east orientations resulted in successful magnetoclinic migratory routes for the autumn migration of juveniles Sharp-tailed Sandpipers from Alaska to New Zealand.

The sun compass was never valid for the spring migration cases (Table 1); still, it seems that the sun compass is useful for long-lasting flights starting from high latitudes (Alerstam et al. 2001), but here only within a limited range of latitudes (61–69°N). At high latitudes, the longitudes are crossed with higher speed causing the route direction to quickly change and consequently resulting in routes very similar to orthodromic routes (Alerstam and Pettersson 1991). We lack route simulations starting from a substantial range of latitudes further to the south, which would be necessary to include to fully evaluate what routes birds may follow at lower latitudes, and what compass mechanism/-s could explain their flight trajectories across latitudes.

The geographic and magnetic loxodromes have very poor support from the performed simulations for any geographical area (Table 1). A possible explanation is that there are very few simulations for the spring migration, and thus, it is not clear if different compasses are used for the two migratory seasons. An alternative consideration would be that the geographic direction obtained from the star compass is a complementary mechanism used in combination with another compass such as, for example, the magnetoclinic route outlined above, where an additional cue should provide the westerly or easterly direction. However, the stars are almost impossible to see during the polar summer, so they are likely used only later in the season at high latitudes or predominantly at lower latitudes (see also above; Åkesson et al. 1995, 2001a, 2005). Finally, the magnetic loxodrome is the least successful mechanism performing badly in numerousness cases. The magnetic loxodrome is based on a compass that can discriminate geomagnetic north based on the angle of inclination (Wiltschko and Wiltschko 1972). Most of the simulations included here were performed in the northern hemisphere, where the magnetic compass may be challenging to use due to the steepness of the geomagnetic field lines (Wiltschko and Wiltschko 1972). Experimental data from studies performed with wild birds in the field, however, suggest that birds have the capacity to use the natural geomagnetic field to find meaningful migration directions also in the high Arctic (Sandberg et al. 1998; Åkesson et al. 1995, 2001a, 2005; Muheim et al. 2006), including waders (Gudmundsson and Sandberg 2000; Grönroos et al. 2010).

### Do we find differences in compass use between geographic regions?

The available studies mainly focus on autumn migration in the North American migration system with starting locations at high latitudes (>66°N). The simulations from this region, however, include both passerine and wader species (Table 1). If we exclude the special case of waders from Baffin Island (where all compass mechanism resulted valid, see above), the only hypothetical mechanisms valid are the sun compass and the magnetoclinic route (Table 1). However, in addition, the sun compass is only valid for waders but never for passerines, whereas the magnetoclinic hypothesis resulted in realistic routes for both passerines and waders in this region (Table 1). This is in line with the fact that a sun compass not compensating for the longitudinal time shift works for long continuous flights, such as the ones performed by arctic waders. Tracking data for some species of shorebirds inhabiting the arctic tundra show that they may not stop as frequently as passerines on autumn migration (e.g., Watts et al. 2008; Gill et al. 2009; Klaassen et al. 2011). Indeed, during the stop-over period, the internal clock of the bird will be reset to the local stop-over time, which may take approximately 3 days and nights (Schmidt-Koenig 1990), and as a consequence, the complete route will likely have less “curved” shape and consequently lead to a different destination. All simulations done so far (Alerstam et al. 2001; Grönroos et al. 2010; Åkesson and Bianco 2016) have been performed for continuous flight not allowing the bird to stop and consequently reset its internal clock. As explained above for the case of the Northern Wheatear, a sun compass compensating for the longitudinal displacement would result in routes similar to loxodromes with almost constant geographic direction.

Other geographical areas have been less studied than the North America, and in the Palaearctic-African migration system, the results are less unequivocal, while for the migration across the Pacific, the magnetoclinic route is the only route in all cases compatible with the predicted migratory flyway (Åkesson and Bianco 2016). Probably, during such a long flight across the ocean, a compass that is available all across the route and in different visibility conditions will be needed. The geomagnetic field provides this information (Fig. 2), but how birds may keep track of their own movement relative to the ground and the geomagnetic field under those conditions remains to be explained. The flight, furthermore, crosses the magnetic equator (Gill et al. 2009; Åkesson and Bianco 2016), and in theory, the magnetoclinic route should not be affected by this passage (Wiltschko and Wiltschko 1972, 1992), since the avian magnetic compass is based on the angle of inclination used in this mechanism. The only consequence of crossing the magnetic equator (and the inversion of the angle of inclination of the magnetic field) is that a bird flying from north to south will change course from south-east to south-west or south-west to south-east and vice versa if the migration direction was southward (Kiepenheuer 1984). A sun compass course will be affected by an equator passage, as it needs to change the course relative to the sun based on an additional mechanism because of the change in the sun’s path across the sky which is different north and south of the equator (see problems outlined at equator regions above).

### Orientation in relation to topography and winds

All simulations in Table 1 assume that the compass direction along the entire route is fixed for all four compass mechanisms, and the simulations are performed relative to the end goal at the wintering area or the breeding area, respectively, without considering the possibility of an intermediate goal (Rabøl 1978; Willemoes et al. 2014), or navigation using a map and a compass (Kramer 1957). In reality, terrestrial bird migrants regularly spend time at stopover for refuelling along the route (e.g., Schmaljohann et al. 2012; Willemoes et al. 2014), while only in extreme situations, they are forced to engage in long continuous flights (e.g., Gill et al. 2009; Klaassen et al. 2011; Åkesson et al. 2016). In the simulations, birds are, furthermore, assumed to respond only to cues affecting their compass mechanisms (e.g., the geomagnetic field, stars, and the sun), and not to other local topography such as coastlines and mountain ridges that could affect their orientation. Coastlines could be used as visual cues for navigation and wind drift compensation at low flight altitudes (e.g., Åkesson 1993b; Bruderer and Liechti 1998; Hedenström and Åkesson 2016), while ecological barriers could require a change of strategy such, as, for example, crossing deserts in the fastest possible way (e.g., Åkesson et al. 2016), or by performing detours to avoid large inhospitable regions such as oceans or large mountains (e.g., Gudmundsson et al. 1995).

Birds are largely affected by winds on migration (Evans 1966; Alerstam 1976), as winds regularly reach the same or higher speeds than the air speeds of birds (e.g., Liechti and Bruderer 1998; Pennycuick et al. 2013). Strong sidewinds may further cause birds to drift off intended courses during natural migrations (Alerstam 1979; Klaassen et al. 2011). To compensate for wind drift, migratory birds may fly along topographical features such as coastlines, mountain ridges, or above cities at night for part of the journey providing visual contact with the ground below (Åkesson 1993b; Karlsson et al. 2010; Hedenström and Åkesson 2016). Most of the migrations, however, occur at much higher altitudes (>1000 m asl; e.g., Able 1970; Bruderer and Liechti 1998; Zehnder et al. 2001), where they may be less affected by topography (Zehnder et al. 2001; Nilsson et al. 2014). Migratory birds may selectively depart on migration flights in tailwind conditions (Åkesson and Hedenström 2000; Green 2004; Sjöberg et al. 2015; Åkesson et al. 2016), and perform high altitude migration in light tailwind conditions (e.g., Richardson 1978, 1990; Zehnder et al. 2001), as tailwinds are expected to reduce the transportation cost substantially depending on level of support from the wind (e.g., Alerstam 1976; Richardson 1978). Tailwinds might even be a prerequisite for successful migration in some bird migration systems (Piersma and Jukema 1990; Butler et al.^1997^, Green et al. 2004; Gill et al. 2014; but see Hedenström and Weber 1999). There are also suggestions that birds may sense the direction of wind without visual cues (Demong and Emlen 1978; cf. Chapman et al. 2015), but if so exactly what mechanism may be involved needs to be explained.

In route simulations assessing vector navigation, winds have only recently been considered (Åkesson and Bianco 2016), suggesting that birds likely are capable of compensating for wind drift during continuous migration flights. Birds may change their flight heading to compensate for drift caused by sidewinds, but how birds are able to detect their own movements relative to ground as a result of their own air speed and the effect of winds often at substantial altitudes, however, needs further attention (Hedenström and Åkesson 2017).

### Conclusions and future research

Here, we report that even though the best support was given to the magnetoclinic route as proposed by Kiepenheuer (1984), there is no single compass mechanism that could explain all cases for which alternative routes were simulated including this mechanism. The magnetoclinic route was the only mechanism that found support across the full range of latitudes (1°S–74°N), while the sun compass only worked within the arctic region (61°–69°N; Fig. 3), suggesting potential for wide use across the globe in the former case. Although the magnetoclinic route did not work in a few cases, including the displaced songbirds in the very far north, none of the other mechanisms did either for the displaced birds. The other case where the magnetoclinic route did not work was for two of the sites in the very high arctic from where waders were tracked by radar (Alerstam et al. 2001) because of the difficulties related to the steepness of the geomagnetic field lines at these sites.

Even if we find that the magnetoclinic route could explain a majority of cases across latitudes, we note that to fully understand what compass is used across latitudes and seasons additional data will be needed. Especially, we lack data from latitudes nearer to the equator. Most of the studies performed so far have focused on autumn migration (Alerstam et al. 2001; Muheim et al. 2003; Grönroos et al. 2010; cf. Åkesson and Bianco 2016), and therefore, in this study, we could not evaluate if the same mechanism was used during (the usually faster) spring migration and slower autumn migration. Therefore, we suggest that future studies should address vector navigation during spring migration, and include regions near to the equator. We also propose that more advanced navigation mechanisms including intermediate goals (e.g., Rabøl 1978) and including the use of gradients for map navigation (Kramer 1957; Wallraff 1991) may be considered in future studies. There is also a need to include effects of winds on route trajectories in future simulations, as the ability to compensate for wind drift has been pointed out as a likely component of the most realistic flight routes in previous simulations (Åkesson and Bianco 2016), meaning that the birds potentially are able to keep track of their own movements relative to ground during migration (cf. Hedenström and Åkesson 2017). We, furthermore, acknowledge the limitations in the methods used to evaluate the effect of route simulations so far (Alerstam et al. 2001; Muheim et al. 2003; Åkesson and Bianco 2016, this study), where there is a need to include error metrics in evaluating the success of simulated routes explaining flight trajectories recorded by tracking technology. To be able to compare data across registration methods, we have not included this here, but we propose that future studies should include such error metrics in the evaluation process.

## Electronic supplementary material

Below is the link to the electronic supplementary material.

Supplementary material 1 (PDF 5274 kb)
