# Supplementary material for: Route simulations, compass mechanisms and long-distance migration flights in birds
Source: J Comp Physiol A Neuroethol Sens Neural Behav Physiol. 2017 May 12;203(6):475–90. doi: 10.1007/s00359-017-1171-y (PMC5522512; doi:10.1007/s00359-017-1171-y)
Supplement: Supplementary file 1 — Supplementary material 1 (PDF 5274 kb) [file 359_2017_1171_MOESM1_ESM.pdf]

Supplementary material

Route simulations, compass mechanisms and long-distance migration flights in birds

Susanne Åkesson and Giuseppe Bianco

We simulated migratory routes following the magnetoclinic hypothesis proposed by Kiepenheuer (1984) for the studies which did not include this alternative compass mechanism in previous work (i.e., Alerstam et al. 2001, Muheim et al. 2003, Grönroos et al. 2010). We used the same initial geographic departure direction that was used for each study case and determined the initial apparent inclination angle and the successive route directions using equations 1 and 2 in Kiepenheuer (1984), respectively. The routes' directions were calculated every 10 km and plotted in R ver. 3.2.2 (R Core Team 2016) using the packages *geosphere* 1.5-5 and *maps* 3.1.1. The geomagnetic parameters at each location were obtained from the Fortran code of the 12th generation of the International Geomagnetic Reference Field model (IGRF-12; Thébault et al. 2015) available at www.ngdc.noaa.gov/IAGA/vmod/igrf.html for the median date of the orientation data provided for each case (references in the main text, Table 1).

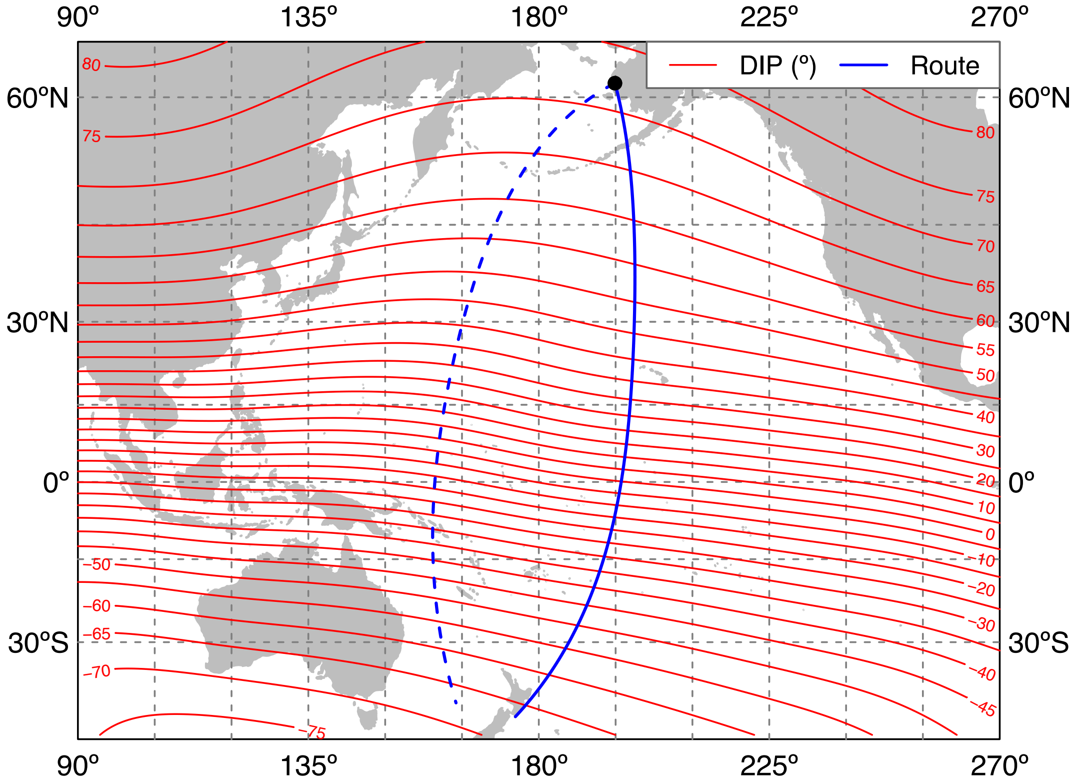

Figure S1. Simulated magnetoclinic routes for the autumn migration of juveniles Sharp-tailed Sandpipers (*Calidris acuminata*) from the staging area in southwest Alaska for mean directions recorded by orientation cage experiments under overcast (dashed line) and clear sky (solid line) conditions (Grönroos et al. 2010). Routes are 12000 km long and their destination is compatible with the wintering area for the species in New Zealand. Map is in Mercator projection with 15° grid and reports the isolines of magnetic inclination (DIP) at 5° intervals.

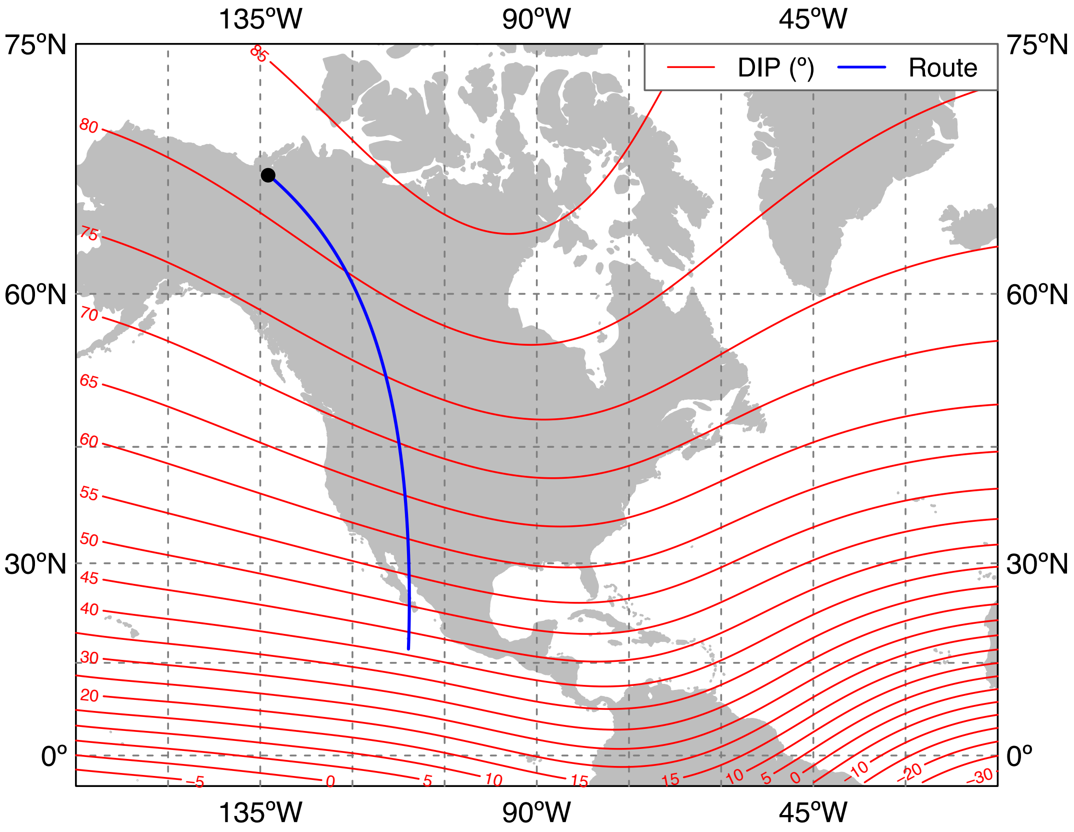

Figure S2. Simulated magnetoclinic route for the autumn migration of Savannah Sparrow (*Passerculus sandwichensis*) from Inuvik (Canada). Initial direction given is generated by orientation cage experiment under overcast conditions (Muheim and Åkesson 2002). Route is 6000 km long and compatible with the wintering area destination for the species in western United States and Mexico. Map is in Mercator projection with 15° grid and reports the isolines of magnetic inclination (DIP) at 5° intervals. Routes with alternative compass mechanisms are reported in Muheim et al. (2003).

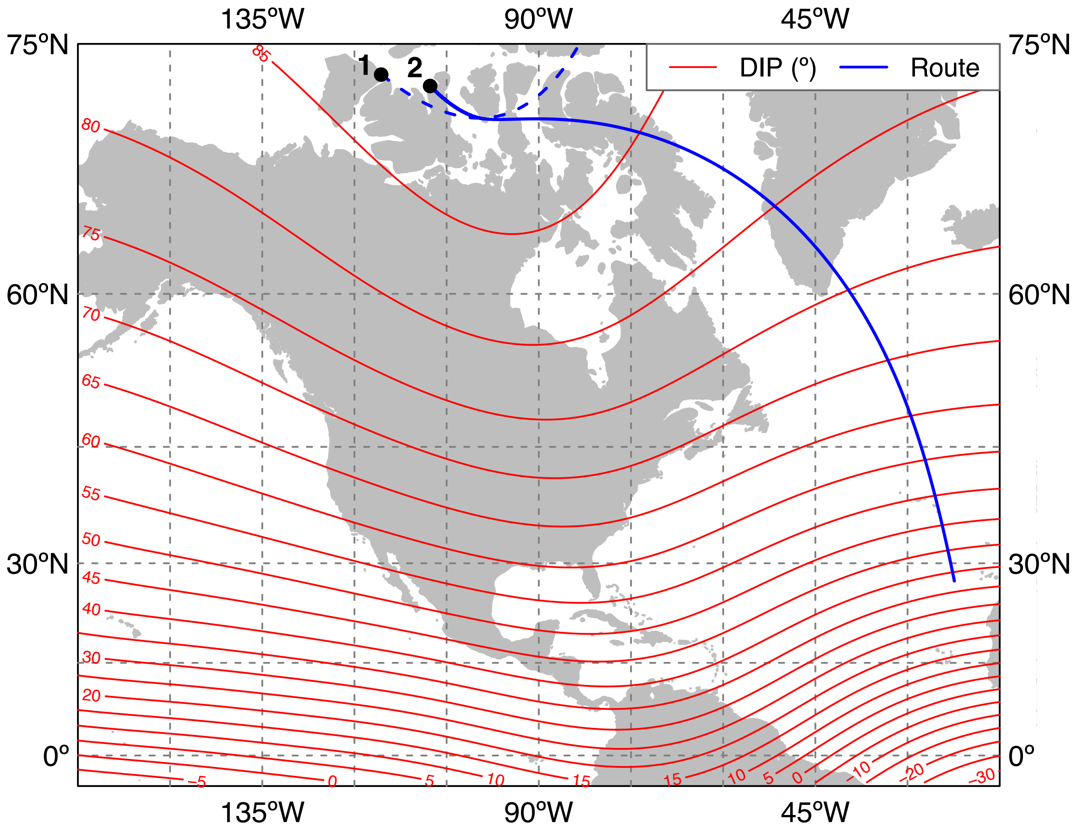

Figure S3. Simulated magnetoclinic routes for the autumn migration of White-crowned Sparrows (*Zonotrichia leucophrys*) captured at Inuvik (Canada) and displaced to (1) Banks Island and (2) Melville Island. Initial directions are from orientation cage experiments under overcast conditions (Åkesson et al. 2001). Both routes are not compatible with the expected wintering area of this species in southwestern United States with route from location 1 being an extreme scenario with the apparent inclination angle being equal to the starting location inclination value and thus the magnetoclinic route is heading toward north following the starting location isocline. Map is in Mercator projection with 15° grid and reports the isolines of magnetic inclination (DIP) at 5° intervals. Routes with alternative compass mechanisms from the same sites are reported in Muheim et al. (2003).

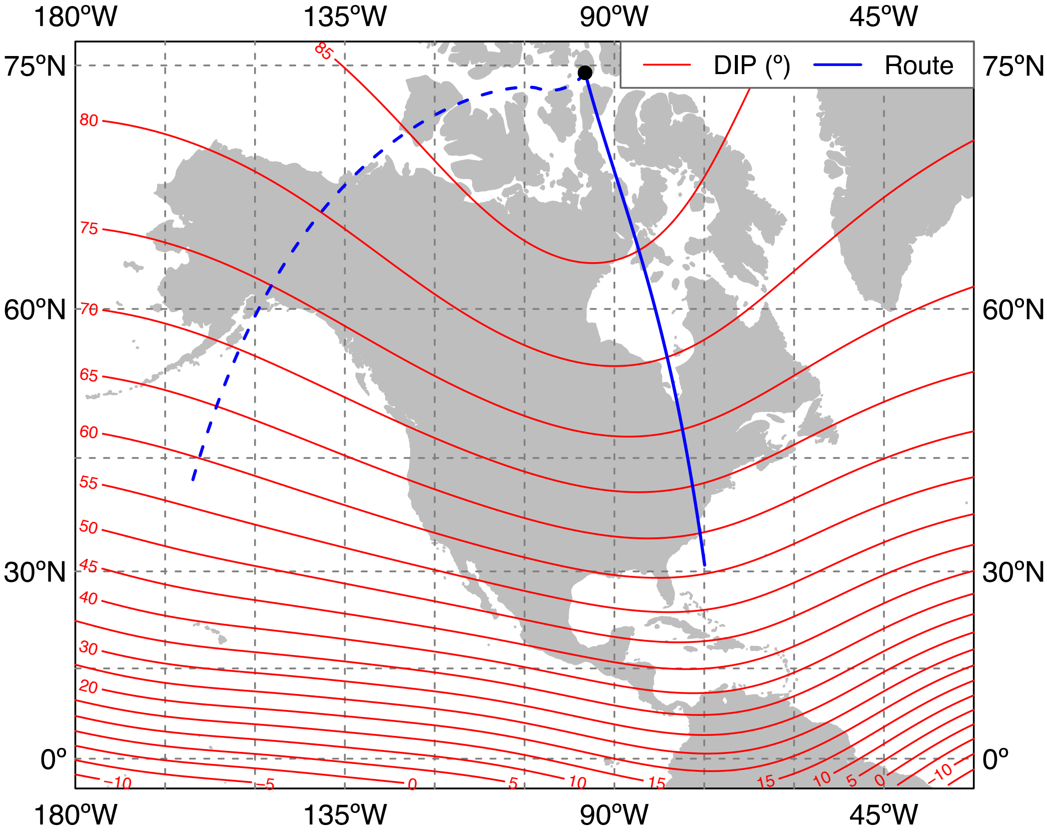

Figure S4. Simulated magnetoclinic routes for the autumn migration of Snow Bunting (*Plectrophenax nivalis*) from Resolute (Cornwallis Island). Initial directions are from orientation cage experiment under simulated overcast conditions (dashed line) and from release experiment under natural overcast conditions (solid line; Sandberg et al. 1998). Simulations are based on preferred orientation generated for fat birds, which was also used in Muheim et al. (2003), to make the alternative routes strictly comparable. Routes are 5000 km long and only the results from release experiment orientation is compatible with the wintering area for the species in eastern United States. Map is in Mercator projection with 15° grid and reports the isolines of magnetic inclination (DIP) at 5° intervals. Routes with alternative compass mechanisms are reported in Muheim et al. (2003).

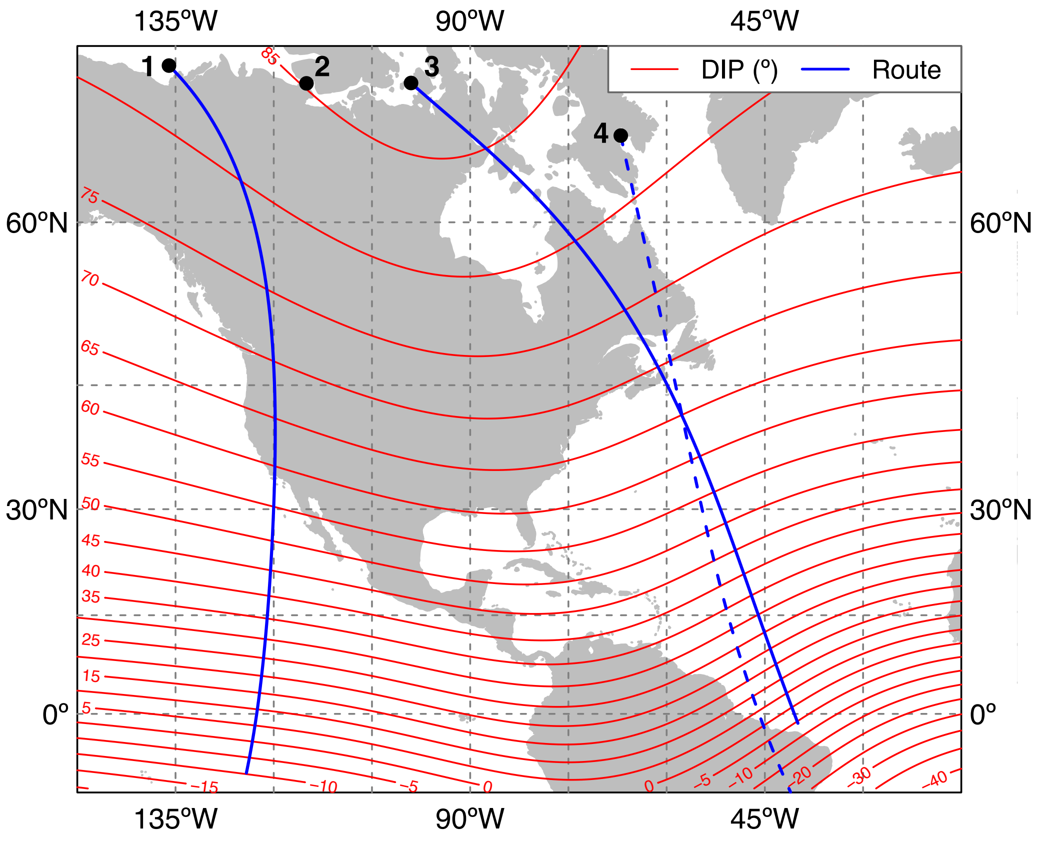

Figure S5. Simulated magnetoclinic routes for the autumn migration of shorebirds from (1) Beufort Sea, (2) Wollaston Peninsula, (3) King William Island, and (4) Baffin Island. Initial directions are from tracking radar data (Alerstam et al. 2001) and route lengths are 9000 km. From location 1 only one geographic direction was compatible with a magnetoclinic route (among the 5 arbitrarily chosen directions in Alerstam et al. (2001). Also for location 2 the direction is not compatible with a magnetoclinic route (see main text for explanation) and is reported only for comparison with maps in Alerstam et al. (2001). Routes from locations 3 and 4 are heading toward the wintering area for those species (South America) and are very similar to some successful routes presented in Alerstam et al. (2001). Map is in Mercator projection with 15° grid and reports the isolines of magnetic inclination (DIP) at 5° intervals.

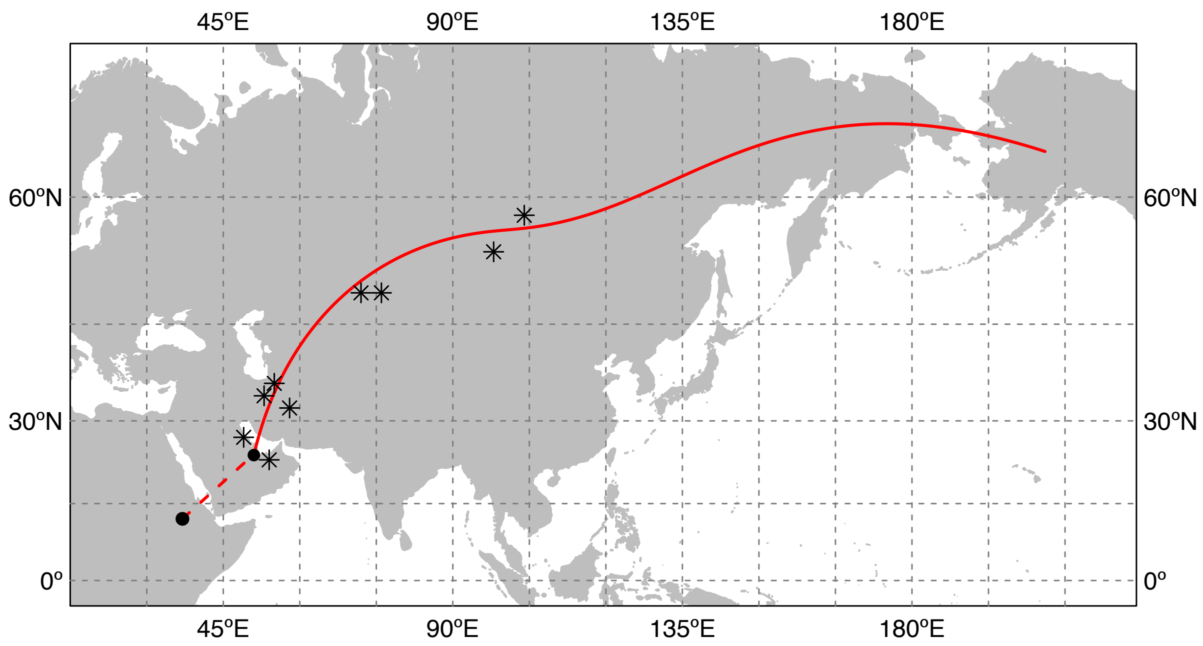

Figure S6. Simulated magnetoclinic routes for the spring migration of Northern Wheatear from eastern Africa to western Alaska. In this example the bird crosses the Red Sea and Arabian Peninsula (dashed line) to an intermediate location before departing for migration following a magnetoclinic route (solid line, 11000 km). Black stars are known stopover locations for this species (Schmaljohann et al. 2012). Map is in Mercator projection with 15° grid.

References

Alerstam T, Gudmundsson GA, Green M, Hedenström A (2001) Migration along orthodromic sun compass routes by arctic birds. Science 291:300-303.

Kiepenheuer J (1984) The magnetic compass mechanism of birds and its possible association with the shifting course directions of migrants. Behavioral Ecology and Sociobiology 14: 81-99

Grönroos J, Muheim R, Åkesson S (2010) Orientation and autumn migration routes of juvenile sharp-tailed sandpipers at a staging site in Alaska. J Exp Biol 213:1829-1835

Muheim R, Åkesson S, Alerstam T (2003) Compass orientation and possible migration routes of passerine birds at high arctic latitudes. Oikos 103:341–349

R Core Team (2016) R: A language and environment for statistical computing. R Foundation for Statistical Computing, Vienna, Austria. URL https://www.R-project.org/

Schmaljohann H, Fox JW and Bairlein F (2012) Phenotypic response to environmental cues, orientation and migration costs in songbirds flying halfway around the world. Anim Behav 84: 623—640

Thébault E, Finlay CC, Beggan CD, Alken P, Aubert J, Barrois O, Bertrand F, Bondar T, Boness, A, Brocco L, Canet E (2015) International geomagnetic reference field: the 12th generation. Earth, Planets and Space, 67: 1-19
